# Predicting the risk of heart failure after acute myocardial infarction using an interpretable machine learning model

**DOI:** 10.3389/fcvm.2025.1444323

**Published:** 2025-01-24

**Authors:** Qingqing Lin, Wenxiang Zhao, Hailin Zhang, Wenhao Chen, Sheng Lian, Qinyun Ruan, Zhaoyang Qu, Yimin Lin, Dajun Chai, Xiaoyan Lin

**Affiliations:** ^1^ Department of Ultrasound, The First Affiliated Hospital, Fujian Medical University, Fuzhou, China; ^2^ National Regional Medical Center, Binhai Branch of the First Affiliated Hospital, Fujian Medical University, Fuzhou, China; ^3^ Department of Cardiology, The First Affiliated Hospital, Fujian Medical University, Fuzhou, China; ^4^ Fujian Key Laboratory of Network Computing and Intelligent Information Processing, College of Computer and Data Science, Fuzhou University, Fuzhou, China; ^5^ Key Laboratory of Metabolic Cardiovascular Disease of Fujian Province Colleges and Universities, Fuzhou, China; ^6^ Clinical Research Center for Metabolic Heart Disease of Fujian Province, Fuzhou, China

**Keywords:** acute myocardial infarction, heart failure, machine learning, predict, shapley additive explanations

## Abstract

**Background:**

Early prediction of heart failure (HF) after acute myocardial infarction (AMI) is essential for personalized treatment. We aimed to use interpretable machine learning (ML) methods to develop a risk prediction model for HF in AMI patients.

**Methods:**

We retrospectively included patients initially with AMI who received percutaneous coronary intervention (PCI) in our hospital from November 2016 to February 2020. The primary endpoint was the occurrence of HF within 3 years after operation. For developing a predictive model for HF risk in AMI patients, the least absolute shrinkage and selection operator (LASSO) Regression was used to feature selection, and four ML algorithms including Random Forest (RF), Extreme Gradient Boost (XGBoost), Support Vector Machine (SVM), and Logistic Regression (LR) were employed to develop the model on the training set. The performance evaluation of the prediction model was carried out on the training set and the testing set, utilizing metrics including AUC (Area under the receiver operating characteristic curve), calibration plot, and decision curve analysis (DCA). In addition, we used the Shapley Additive Explanations (SHAP) value to determine the importance of the selected features and interpret the optimal model.

**Results:**

A total of 1220 AMI patients were included and 244 (20%) patients developed HF during follow-up. Among the four evaluated ML models, the XGBoost model exhibited exceptional accuracy, with an AUC value of 0.922. The SHAP method showed that left ventricular ejection fraction (LVEF), left ventricular end-systolic diameter (LVDs) and lactate dehydrogenase (LDH) were identified as the three most important characteristics to predict HF risk in AMI patients. Individual risk assessment was performed using SHAP plots and waterfall plot analysis.

**Conclusions:**

Our research demonstrates the potential of ML methods in the early prediction of HF risk in AMI patients. Furthermore, it enhances the interpretability of the XGBoost model through SHAP analysis to guide clinical decision-making.

## 1 Introduction

Despite the progress in percutaneous coronary intervention (PCI), myocardial infarction remained a critical condition with high mortality and morbidity (1). Heart failure (HF) after acute myocardial infarction (AMI) was the main cause of increased mortality in patients with AMI (2). According to the research report, HF after AMI increased the risk of death of patients by 3–4 times (3). Therefore, early identification of the risk of HF after myocardial infarction and the implementation of personalized treatment can result in a reduction in mortality and an enhancement of quality of life for patients.

Currently, predictive models for evaluating the prognosis of AMI patients were primarily constructed using logistic regression (LR) methods. Commonly risk scoring systems, such as the Thrombolysis in Myocardial Infarction (TIMI) Risk Score (4) and the Global Registry of Acute Coronary Events (GRACE) Risk Score (5), However, these prognostic models had some limitations (6). First, these systems rely only on traditional risk factors, such as age, smoking, hypertension, and diabetes, these systems relied only on traditional risk factors, such as age, smoking, hypertension, and diabetes, and did not include key prognostic indicators such as laboratory data and echocardiographic parameters (7, 8), which might not adequately reflect the multiple and complex pathophysiological processes that lead to the development and progression of AMI (9). Additionally, these models were mainly used to predict mortality, and their accuracy in predicting heart failure was limited.

Machine learning (ML) models have been shown to improve risk prediction in various cardiovascular disease (10, 11) Through simulating human learning activities, ML automatically obtained information from big clinical data for learning (12, 13). In patients with AMI, utilization of data-driven models to determine the risk of HF has been attempted, Li et al. successfully developed an ML model for predicting the risk of HF after AMI (14), but their study was limited to clinical tests and did not include imaging, and other findings. In addition, the inherent “black box” nature of ML algorithms makes their internal prediction process difficult to interpret, limiting practical applications (15). Therefore, our study introduced the Shapley Additive Explanations (SHAP) method, a game theory-based interpretable ML method developed by Lundberg and Le (16). This method can elucidate the complex relationship between features and predictions. Compared to other explanatory methods mentioned in previous literature, SHAP offered significant advantages in interpretability and visualization, thereby enabling a more comprehensive understanding and interpretation of complex models (17).

Therefore, this study aimed to establish and validate an interpretable ML model for predicting the risk of HF in AMI patients, and used the SHAP method to visualize how the ML model makes decisions. This effective computer-assisted approach can assist frontline clinicians in the early identification and intervention of HF occurrences.

## 2 Materials and methods

### 2.1 Study design and participants

We retrospectively included patients diagnosed with AMI who underwent PCI at the First Affiliated Hospital of Fujian Medical University from November 2016 to February 2020. The inclusion criteria for AMI patients were: (a) age >18 years; (b) according to the current diagnostic guidelines for AMI, the first diagnosis on admission included clinical symptoms, typical changes in the electrocardiogram, and elevated cardiac biomarkers (18), (c) without a history of HF. Patients meeting any of the following exclusion criteria were excluded: history of PCI or coronary artery bypass graft surgery; death during follow-up; moderate to severe valvular heart disease; severe immunological disorders; malignant tumors combined with malignant hematological diseases; severe infections; patients with more than 20% missing data or lost to follow-up.

According to relevant studies and clinical availability, we collected 45 variables related to the risk of HF from electronic medical records, including baseline demographics, clinical comorbidities, laboratory tests, echocardiographic parameters, and angiographic findings (Table 1). All variables were collected within 24 h of admission and immediately before PCI. As some patients underwent emergency PCI, the echocardiography records included data both before and after the procedure.

**Table 1:** Baseline clinical characteristics of the study sample.

Characteristics	Total	Non-HF	HF	*P*-value
	(*n* = 1,220)	(*n* = 976)	(*n* = 244)	
Demographic characteristics				
Age, years	65.0 (57.0–73.0)	65.5 (57.0–72.0)	64.5 (54.0–74.3)	0.758
Gender				0.014
Female	224 (18.4)	198 (20.3)	26 (10.7)	
Male	996 (81.6)	778 (79.7)	218 (89.3)	
Heart rate, beats/mim	78.0 (68.0–90.0)	76.5 (101.0–146.8)	83.0 (72.0–97.3)	<0.001
Systolic blood pressure, mmHg	128.0 (112.0–146.0)	128.0 (112.0–150.0)	119.5 (107.5–135.3)	<0.001
Diastolic blood pressure, mmHg	75.0 (68.0–84.0)	74.0 (68.0–84.0)	76.0 (68.0–86.0)	0.427
Admission diagnosis, STEMI	998 (81.8)	791 (81.0)	207 (84.8)	0.398
Cardiovascular risk factors				
Hypertension	736 (60.3)	588 (60.2)	148 (60.7)	0.934
Diabetes mellitus	364 (29.8)	302 (30.9)	62 (25.4)	0.217
Hypercholesterolemia	462 (37.9)	378 (38.7)	84 (34.4)	0.311
Current smoking	788 (64.6)	632 (64.8)	156 (63.9)	0.866
Alcohol drinking	454 (37.2)	364 (37.3)	90 (36.9)	0.933
Laboratory parameters				
White blood cell count, k/ul	8.8 (6.8–11.3)	8.4 (6.6–10.9)	10.1 (8.0–12.8)	<0.001
Alanine aminotransferase, u/L	32.0 (20.0–55.0)	30.0 (20.0–48.8)	52.0 (24.0–92.5)	<0.001
Aspartate aminotransferase, u/L	57.0 (26.0–152.3)	51.5 (26.0–122.0)	96.0 (24.8–350.8)	0.001
LDH, u/L	333.0 (220.0–659.0)	312.5 (209.5–562.8)	655.0 (250.3–1,180.3)	<0.001
Creatine kinase isoenzyme, u/L	18.0 (12.0–50.0)	16.0 (11.0–42.0)	25.0 (14.0–98.5)	<0.001
Creatinine, umol/L	75.0 (64.0–88.9)	74.0 (63.2–86.7)	82.0 (66.8–99.4)	0.001
Glucose, mmol/L	5.5 (4.8–7.0)	5.4 (4.8–6.9)	5.9 (4.9–7.8)	0.028
Total Cholesterol, mmol/L	4.3 (3.5–4.9)	4.3 (3.5–5.0)	4.3 (3.4–4.8)	0.367
Triglycerides, mmol/L	1.4 (1.0–1.8)	1.4 (0.9–1.8)	1.4 (0.9–1.8)	0.686
High density lipoprotein, mmol/L	1.0 (0.8–1.1)	1.0 (0.8–1.1)	0.9 (0.8–1.1)	0.235
Low density lipoprotein, mmol/L	2.8 (2.1–3.5)	2.9 (2.2–3.5)	2.7 (1.9–3.5)	0.366
Glycated hemoglobin, %	5.9 (5.5–6.7)	5.9 (5.5–6.7)	6.0 (5.6–6.9)	0.223
hsCRP, mg/L	5.7 (1.3–22.2)	5.3 (1.3–18.0)	10.8 (2.2–48.3)	0.001
NT-proBNP, pg/ml	805.0 (295.3–1,940.3)	649.0 (242.3–1,687.5)	1,494.0 (702.0–4,265.0)	<0.001
Cardiac troponin I, ng/ml	1.9 (0.3–7.8)	1.5 (0.3–7.1)	4.2 (0.2–12.3)	0.018
Culprit vessel				
Left main coronary artery	72 (5.9)	60 (6.1)	12 (4.9)	0.606
Left anterior descending	802 (65.7)	648 (66.4)	154 (63.1)	0.495
Left circumflex coronary artery	540 (44.3)	436 (44.7)	104 (42.6)	0.684
Right coronary artery	618 (50.7)	498 (51.0)	120 (49.2)	0.716
Medications at discharge				
Diuretic	982 (80.5)	782 (80.1)	200 (82.0)	0.598
ACEI/ARB	820 (67.2)	660 (67.6)	160 (65.6)	0.666
B-blocker	1,126 (92.3)	900 (92.2)	226 (92.6)	0.879
Statin	1,212 (99.3)	970 (99.4)	242 (99.2)	0.802
Aspirin	1,058 (86.7)	840 (86.1)	218 (89.3)	0.792
Echocardiographic parameters				
LA dimension, cm	4.0 (3.7–4.3)	3.9 (3.7–4.2)	4.2 (3.9–4.5)	<0.001
LVDd, cm	5.0 (4.7–5.4)	4.9 (4.6–5.2)	5.5 (5.1–5.8)	<0.001
LVDs, cm	3.5 (3.1–3.9)	3.3 (3.0–3.7)	4.3 (3.9–4.7)	<0.001
LVVd, ml	118.8 (101.4–141.3)	113.4 (98.8–131.7)	147.7 (124.4–168.5)	<0.001
LVVs, ml	49.1 (37.0–66.7)	44.1 (34.2–56.3)	81.9 (65.1–100.1)	<0.001
IVST, cm	1.0 (0.9–1.1)	1.0 (0.9–1.1)	1.0 (0.9–1.1)	0.217
LVPWT, cm	0.9 (0.8–1.0)	0.9 (0.8–1.0)	0.9 (0.8–1.0)	0.202
Left ventricular mass, g	171.3 (142.8–206.8)	163.5 (138.6–196.8)	201.8 (169.1–236.3)	<0.001
Stroke volume, ml	66.8 (56.2–77.6)	67.6 (57.0–78.1)	61.6 (51.3–74.1)	0.005
LVFS, %	31.0 (25.1–35.6)	32.4 (28.7–36.3)	20.9 (18.1–25.3)	<0.001
LVEF, %	58.3 (49.3–65.0)	60.4 (55.1–65.9)	42.0 (37.2–49.5)	<0.001
E/e	4.6 (3.9–5.5)	4.6 (3.9–5.4)	4.6 (3.9–5.9)	0.748

LDH, lactate dehydrogenase; hsCRP, hypersensitive C-reactive protein; NT-proBNP, N-terminal pro-B-Type Natriuretic Peptide; ACEI, angiotensin-converting enzyme inhibitor; ARB, angiotensin receptor blocker; LVDd, left ventricular end-diastolic dimension; LVDs, left ventricular end-systolic dimension; LVVd, left ventricular end-diastolic volume; LVVs, left ventricular end-systolic volume; IVST, interventricular septal thickness; LVPWT, left ventricular posterior wall thickness; LVFS, left ventricular fraction shortening; LVEF, left ventricular ejection fraction; E/e, The ratio of peak velocity (E) of early diastolic mitral valve orifice blood flow to peak velocity (e) of annular motion.

### 2.2 Follow-up

The primary endpoint was the development of new HF events (New York Heart Association HF classification from II to IV), including AMI patients who developed HF during hospitalization and within the 3-year follow-up period. Data on each patient were obtained from electronic medical records and then verified through outpatient follow-up and telephone calls. Follow-up ended on 20 March 2023. This study was approved by the Institutional Review Committee of the First Affiliated Hospital of Fujian Medical University [No: MRCTA and ECFAH of FMU (2021)072; March 4, 2021]. Due to the retrospective observational design, the requirement for informed consent was eliminated.

### 2.3 Feature selection, model development and performance evaluation

The dataset was randomly divided into a training set (70%, *N* = 854) and a testing set (30%, *N* = 366). To enhance prediction accuracy and interpretability, we employed the least absolute shrinkage and selection operator (LASSO) regression to select key features from the training set (19). LASSO is a regression method designed for high-dimensional data. It introduces a penalty term to the least squares method, compressing some regression coefficients to zero, which achieves variable selection and improves the model's generalization capability (20). In this study, we used the “glmnet” package in R to perform LASSO regression and optimal lambda parameters were determined using 10-fold cross-validation, with the Lambda.1se value corresponding to the minimum cross-validation error selected as the model's optimal value. The count of variables with non-zero regression coefficients at this optimal value was conducted.

Four ML models were developed using the training sets, including random forest (RF), extreme gradient boosting (XGBoost), support vector machine (SVM), and LR classifiers. All continuous variables were normalized to a distribution with a mean of 0 and a standard deviation of 1. At the same time, to address the impact of data imbalance, we adopted the Synthetic Minority Over Sampling Technique (SMOTE) to improve the final predictive performance of the model (21).

In addition, we used several evaluation metrics on the testing set to assess the performance of different ML models, including the area under the receiver operating characteristic curve (AUC), accuracy, sensitivity, specificity, precision, recall, and F1 score. Calibration curves were used to evaluate calibration capability, and decision curve analysis (DCA) was used to evaluate clinical applicability.

### 2.4 Model interpretation

When applying ML predictive models to clinical decision-making, it is crucial to understand how the model predicts individual risk. Therefore, we utilized the SHAP method to visually interpret the optimal mode (22) and to observe the contribution of features to the model's output at the individual level. By randomly selecting one patient who did not develop HF during follow-up and another who did, we evaluated the contribution of features to individual predictions, providing a tailored risk assessment for each patient.

### 2.5 Statistical analysis

Statistical R software (version 3.6.3) and Python software (version 3.7.0) were used for data analysis, model development, and validation. Continuous variables were represented as the median (p25, p75), whereas categorical variables were represented as numbers (*n*) and proportions (%). Baseline characteristics of groups were compared using the Wilcoxon rank-sum test for continuous variables and the chi-square test for categorical variables, considering *P* < 0.05 as statistically significant.

## 3 Results

### 3.1 Patient characteristics

**Figure 1:**
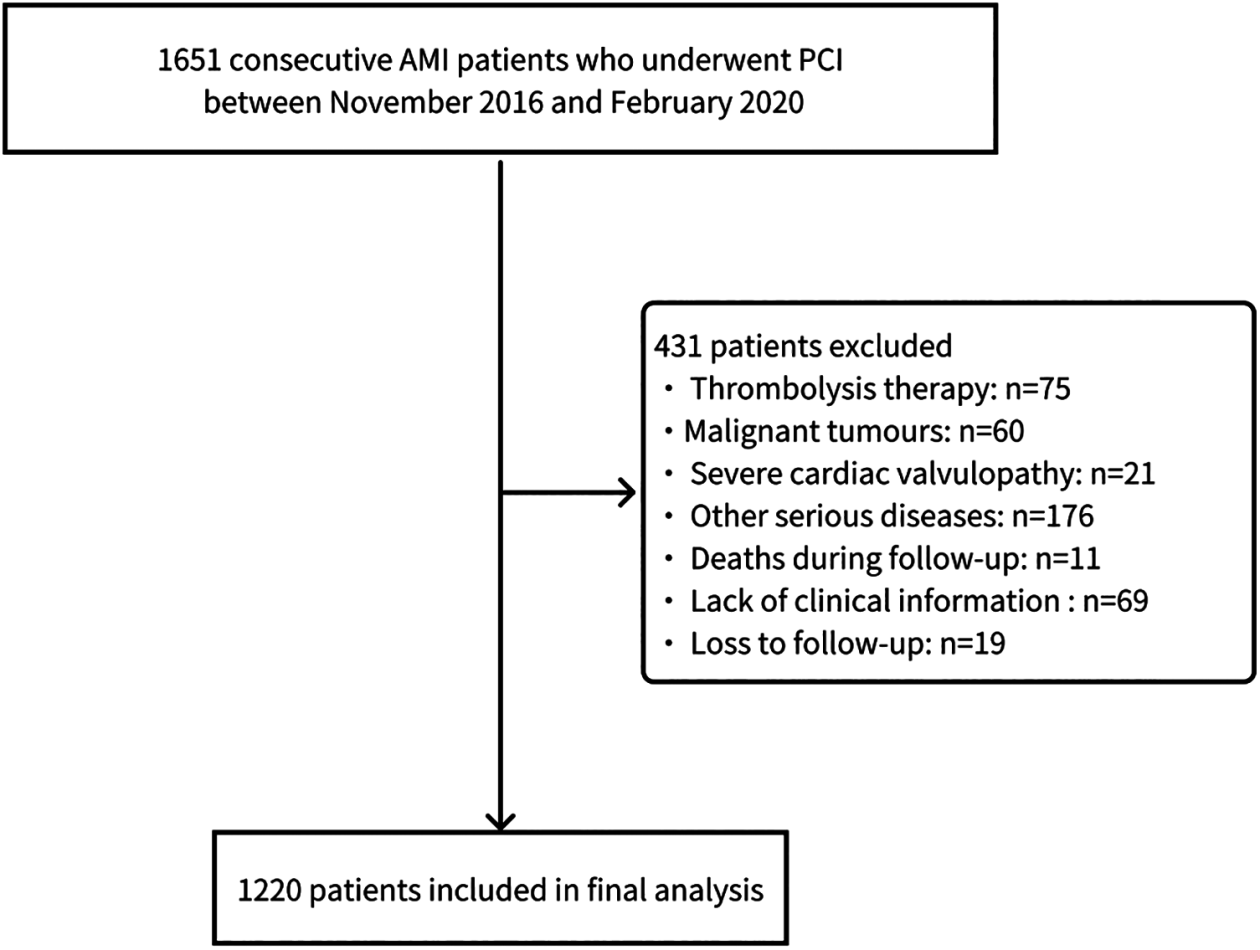
Patient selection flowchart.

The screening process is illustrated in Figure 1. According to the inclusion and exclusion criteria, this study included 1,220 patients with AMI. During the follow-up period, a total of 244 patients (20%) developed HF. Differences in baseline characteristics are summarized in Table 1. Compared to the Non-HF group, the HF group had a higher proportion of male patients (*P* = 0.014), higher heart rates (*P* < 0.001), and lower systolic blood pressure (*P* < 0.001). Baseline levels of white blood cell count, alanine aminotransferase, aspartate aminotransferase, lactate dehydrogenase (LDH), creatine kinase isoenzyme (CK-MB), creatinine, glucose, hypersensitive C-reactive protein (hsCRP), N-terminal pro-B-Type Natriuretic Peptide (NT-proBNP), and cardiac troponin I were significantly elevated in the HF group compared to the Non-HF group (*P* < 0.05). Additionally, the HF group had greater left heart size, left ventricular volume, and left ventricular mass at baseline, whereas stroke volume, left ventricular short-axis shortening rate, left ventricular ejection fraction (LVEF), The ratio of peak velocity (*E*) of early diastolic mitral valve orifice blood flow to peak velocity (*e*) of annular motion were lower (*P* < 0.05). The specific baseline data between the training and testing sets were shown in Table 2, with no significant differences between the two groups (*P* > 0.05).

**Table 2:** Patient characteristics in training and testing sets.

Characteristics	Training	Testing	*P*-value
	(*n* = 854)	(*n* = 366)	
HF	170 (19.9)	74 (20.2)	0.310
Basic characteristics			
Age, years	64.0 (57.0–73.0)	63.5 (58.0–72.5)	0.683
Gender			0.553
Female	162 (19.0)	62 (16.9)	
Male	692 (81.0)	304 (83.1)	
Heart rate, beats/mim	78.0 (69.0–90.0)	78.0 (69.0–90.0)	0.258
Systolic blood pressure, mmHg	128.0 (112.0–146.0)	125.0 (110.0–144.0)	0.104
Diastolic blood pressure, mmHg	76.0 (68.0–85.0)	74.0 (68.0–84.0)	0.516
Cardiovascular risk factors			
Hypertension	518 (60.7)	218 (59.6)	0.800
Diabetes mellitus	224 (26.2)	102 (27.9)	0.675
Hypercholesterolemia	323 (37.8)	139 (38.0)	0.778
Current smoking	544 (63.7)	244 (66.7)	0.483
Alcohol drinking	336 (39.3)	118 (32.2)	0.096
Laboratory parameters			
White blood cell count, k/ul	8.8 (7.1–11.3)	8.7 (6.7–11.3)	0.686
Alanine aminotransferase, u/L	32.0 (20.0–54.0)	33.0 (20.0–56.0)	0.622
Aspartate aminotransferase, u/L	57.0 (25.0–161.0)	56 (26.0–161.0)	0.752
LDH, u/L	339.0 (220.0–659.0)	327.0 (220.0–659.0)	0.915
Creatine kinase isoenzyme, u/L	18.0 (12.0–54.0)	17 (11.0–35.0)	0.154
Creatinine, umol/L	75 (64.0–89.0)	75.8 (64.0–88.0)	0.702
Glucose, mmol/L	5.5 (4.8–6.9)	5.6 (4.8–7.4)	0.478
Total Cholesterol, mmol/L	4.3 (3.5–5.0)	4.3 (3.6–4.9)	0.784
Triglycerides, mmol/L	1.4 (1.0–1.8)	1.3 (0.9–2.0)	0.664
High density lipoprotein, mmol/L	1.0 (0.8–1.1)	1.0 (0.8–1.1)	0.257
Low density lipoprotein, mmol/L	2.8 (2.1–3.5)	2.8 (2.1–3.4)	0.982
Glycated hemoglobin,%	5.9 (5.5–6.7)	5.9 (5.5–6.9)	0.945
hsCRP, mg/L	5.7 (1.4–20.4)	5.9 (1.3–25.0)	0.592
NT-proBNP, pg/ml	744.0 (280.0–2,050.0)	902.0 (354.0–1,860.0)	0.759
Cardiac troponin I, ng/ml	1.9 (0.3–8.5)	1.6 (0.3–5.7)	0.616
Culprit vessel			
Left main coronary artery	92 (10.7)	20 (10.9)	0.811
Left anterior descending	277 (64.9)	124 (67.8)	0.491
Left circumflex coronary artery	192 (45.0)	78 (42.5)	0.594
Right coronary artery	205 (48.0)	104 (56.8)	0.051
Medications at discharge			
Diuretic	351 (82.2)	140 (76.5)	0.079
ACEI/ARB	286 (67.0)	124 (67.8)	0.851
B-blocker	394 (92.3)	169 (92.3)	0.974
Statin	425 (99.5)	181 (98.9)	0.381
Aspirin	367 (85.9)	162 (88.5)	0.481
Echocardiographic parameters			
LA dimension, cm	4.0 (3.7–4.3)	4.0 (3.7–4.4)	0.084
LVDd, cm	5.0 (4.7–5.4)	5.0 (4.7–5.4)	0.649
LVDs, cm	3.4 (3.1–3.9)	3.5 (3.0–4.0)	0.703
LVVd, ml	119.4 (101.9–141.3)	117.7 (100.8–142.5)	0.646
LVVs, ml	48.1 (37.6–65.5)	51.6 (35.9–69.2)	0.706
IVST, cm	1.0 (0.9–1.2)	1.0 (0.9–1.1)	0.994
LVPWT, cm	0.9 (0.8–1.0)	0.9 (0.8–1.1)	0.683
Left ventricular mass, g	169.0 (141.3–206.4)	174.0 (144.7–211.6)	0.359
Stroke volume, ml	67.0 (56.5–77.6)	66.1 (55.7–77.4)	0.703
LVFS, %	31.1 (25.9–35.7)	31.9 (26.8–36.2)	0.306
LVEF, %	58.6(50.8–65.0)	58.8(47.5–64.8)	0.395
E/e'	12.0(9.7–15.3)	12.4(10.0–16.0)	0.493

LDH, lactate dehydrogenase; hsCRP, hypersensitive C-reactive protein; NT-proBNP, N-terminal pro-B-Type Natriuretic Peptide; ACEI, angiotensin-converting enzyme inhibitor; ARB, angiotensin receptor blocker; LVDd, left ventricular end-diastolic dimension; LVDs, left ventricular end-systolic dimension; LVVd, left ventricular end-diastolic volume; LVVs, left ventricular end-systolic volume; IVST, interventricular septal thickness; LVPWT, left ventricular posterior wall thickness; LVFS, left ventricular fraction shortening; LVEF, left ventricular ejection fraction; E/e, The ratio of peak velocity (E) of early diastolic mitral valve orifice blood flow to peak velocity (e’) of annular motion.

### 3.2 Feature selection

**Figure 2:**
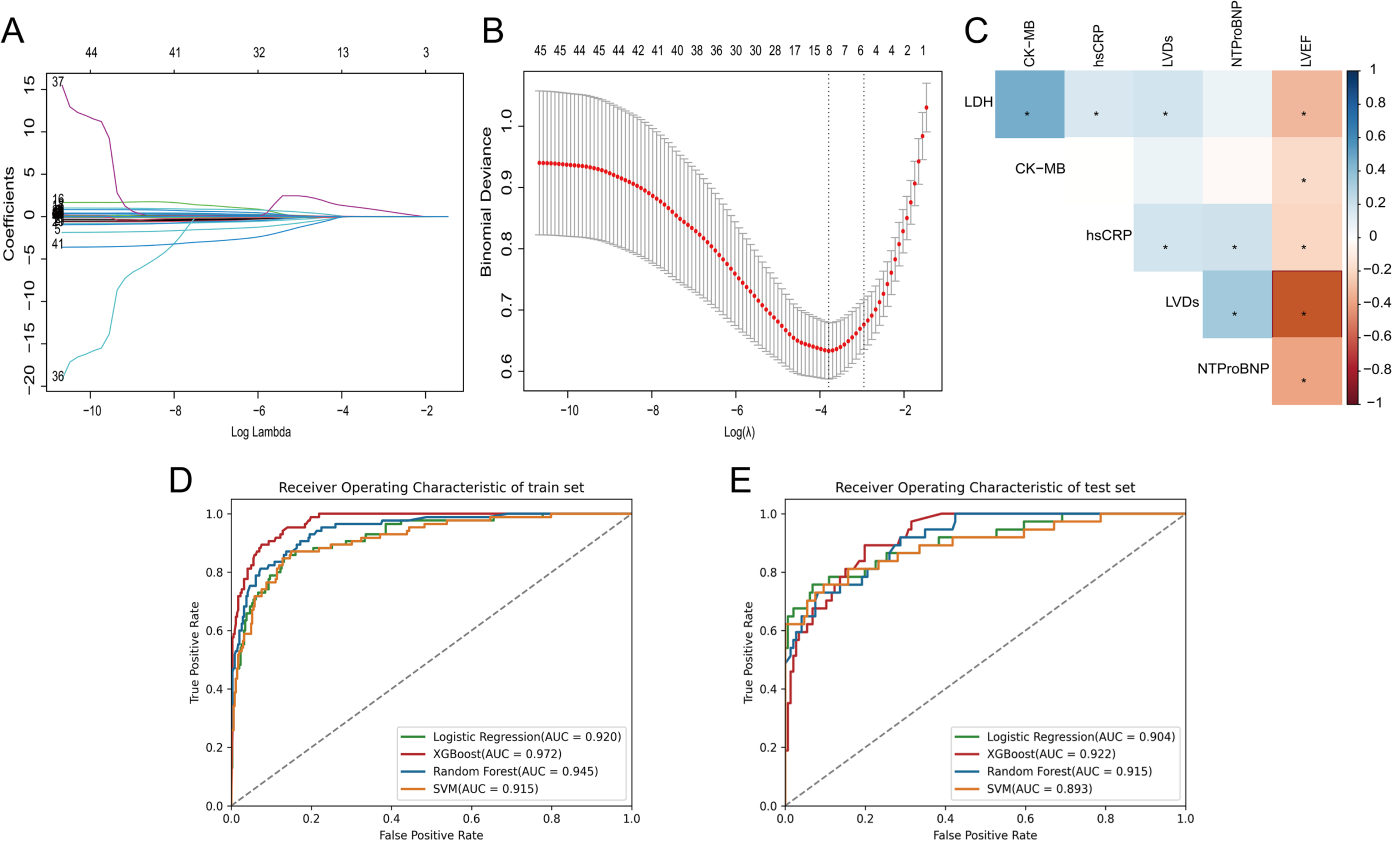
**(A)** The LASSO coefficient profiles of all variables, **(B)** selection of appropriate parameters, **(C)** correlation coefficients between clinical characteristics, **(D)** ROC curves of ML models in training set, **(E)** ROC curves of ML models in testing set.

In this study, six features with non-zero regression coefficients were selected to construct predictive models through LASSO feature selection analysis (Figures 2A,B), such as LDH, CK-MB, hsCRP, NT-proBNP, LVEF and left ventricular end-systolic dimension (LVDs). In addition, the correlation between these features was illustrated by a heatmap based on the matrix of correlation coefficients (Figure 2C). All correlation coefficients were below 0.80, indicating that there was no serious collinearity among the features.

### 3.3 Development and evaluation of models

We used four ML models, RF, XGBoost, SVM, and LR, combined with the above six features to predict the risk of HF after AMI. Figures 2D,E and Table 3 describe the performance of these predictive models, with results indicating that the XGBoost model exhibits better discriminative ability. Compared to other ML models, the XGBoost model exhibited the greatest AUC on both the training and testing set. Although the XGBoost model was slightly lower than the LR model in specificity and F1 score in the testing set, it still outperformed the other models in other performance metrics.

**Table 3:** Performance comparison of the ML models in training and testing sets.

Model	AUC	Accuracy	Sensitivity	Specificity	Precision	Recall	F1-score
Training set							
XGBoost	0.972 (0.957–0.985)	0.901 (0.871–0.930)	0.729	0.974	0.873	0.729	0.795
RF	0.945 (0.918–0.970)	0.906 (0.878–0.934)	0.682	0.962	0.817	0.682	0.744
SVM	0.915 (0.874–0.949)	0.885 (0.855–0.916)	0.635	0.947	0.750	0.635	0.688
LR	0.920 (0.882–0.951)	0.900 (0.865–0.921)	0.659	0.962	0.812	0.659	0.727
Testing set							
XGBoost	0.922 (0.877–0.960)	0.896 (0.862–0.949)	0.795	0.939	0.821	0.676	0.716
RF	0.915 (0.865–0.957)	0.891 (0.852–0.940)	0.649	0.955	0.800	0.649	0.708
SVM	0.893 (0.823–0.933)	0.886 (0.832–0.920)	0.622	0.936	0.786	0.622	0.677
LR	0.904 (0.841–0.949)	0.877 (0.812–0.911)	0.676	0.952	0.781	0.595	0.725

**Figure 3:**
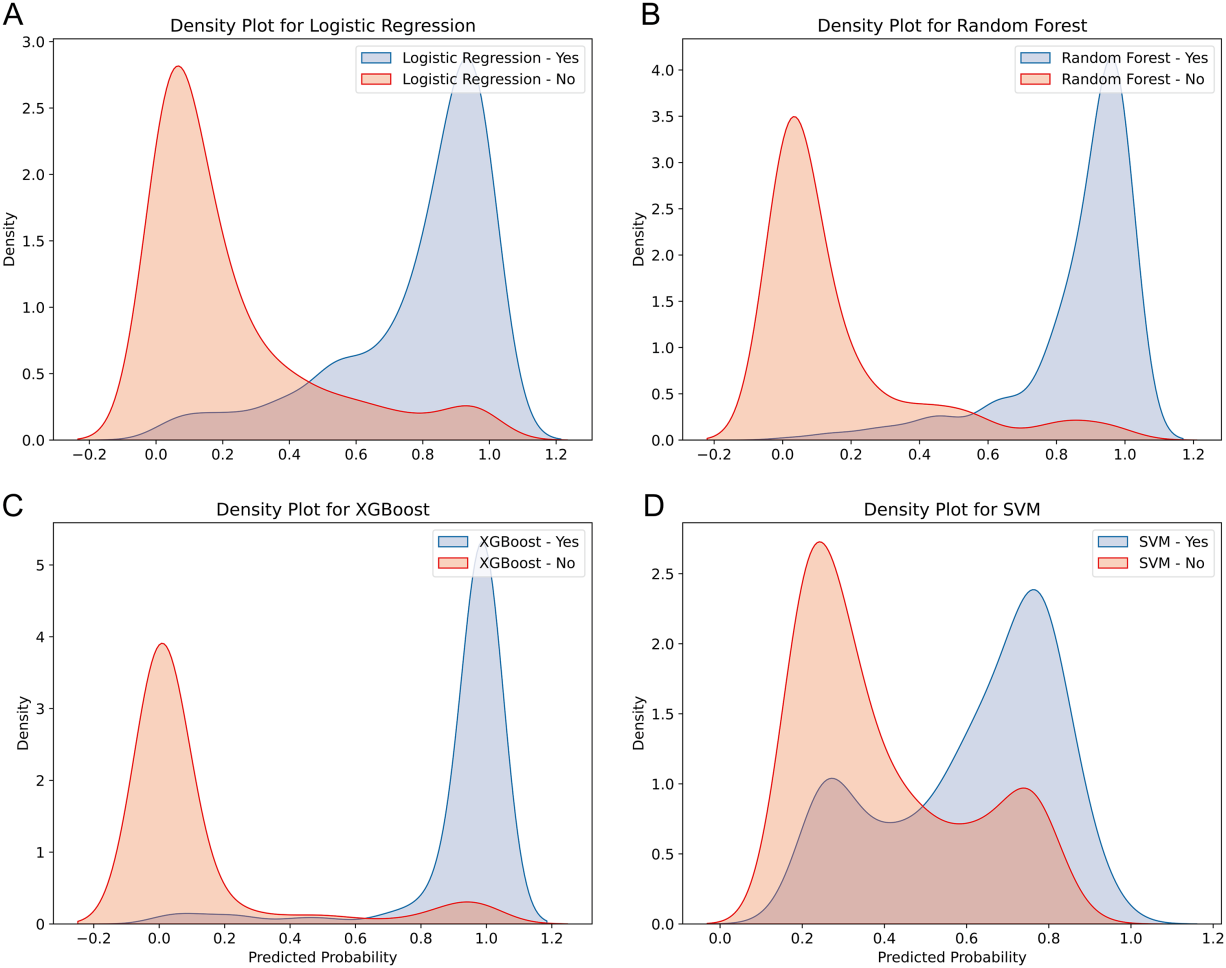
Density curves for all the models. **(A)** LR model, **(B)** RF model, **(C)** XGBoost model, **(D)** SVM model. The orange indicates patients without HF, and the blue indicates patients with HF. The less overlap between the blue and orange colors, the better the model's ability to discriminate.

The discriminative ability of the models in predicting HF after AMI was further analyzed by plotting the density curves (Figures 3A–D). The results showed that the XGBoost model had the smallest overlap and a large discriminative area, followed by LR and RF model, indicating their better discriminative ability. In contrast, SVM model had a relatively large overlap area.

**Figure 4:**
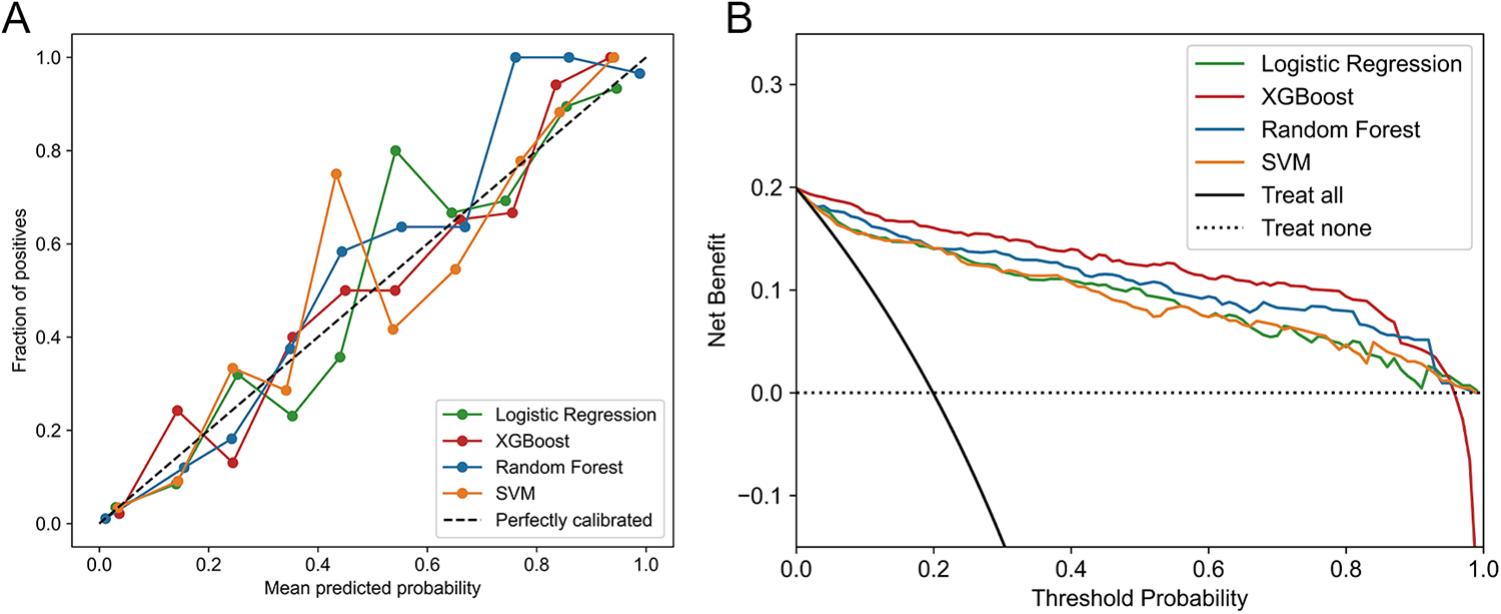
**(A)** Calibration curves of ML models in testing set. **(B)** DCA of ML models in Testing set.

Furthermore, XGBoost's calibration curve closely approximated the ideal line (Figure 4A). DCA was shown in Figure 4B, where the XGBoost model had the highest net benefit when the threshold probability was in the range of 0%-95%. Therefore, based on above findings, the XGBoost model was considered the optimal prediction model.

### 3.4 Model interpretation

**Figure 5:**
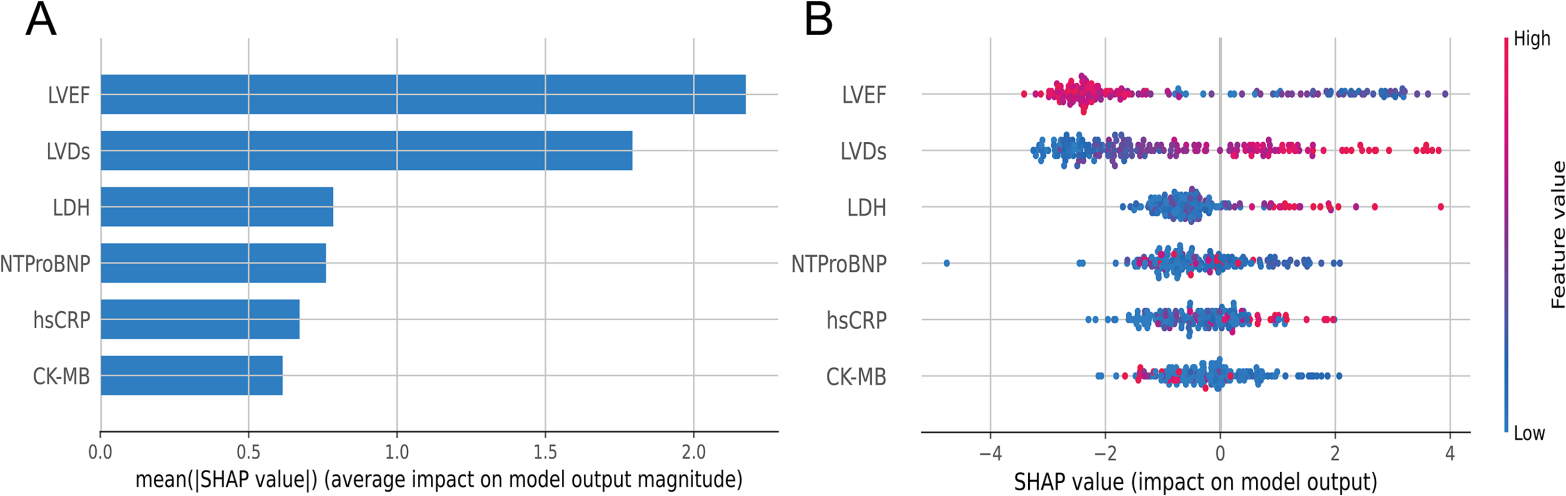
**(A)** Summary plots of SHAP values. Ranking of variable importance based on the average value. **(B)** Representation of the influence exerted by each feature on the final model output, assessed via SHAP values distribution. Every individual patient is denoted by a data point within each row. The red dots represent higher feature values, while the blue dots represent lower feature values. A higher SHAP value indicates a higher HF risk.

To visually interpret the selected features, we used SHAP analysis to interpret the ML model. At the feature level, we used SHAP summary plots to show how these features affected the probability of HF (Figure 5). Figure 5A shows the ranking of feature importance based on Shapley values, which indicated that the three most important features contributing to the prediction model were LVEF, LVDs, and LDH. Figure 5B provided a comprehensive visualization of how the features affected the XGBoost model, where red represented high-risk values and blue represented low-risk values. As seen from the figure, a higher LVEF value (red points) correlated with a lower likelihood of developing HF. Conversely, higher values of LVDs and LDH are associated with higher risk of HF.

**Figure 6:**
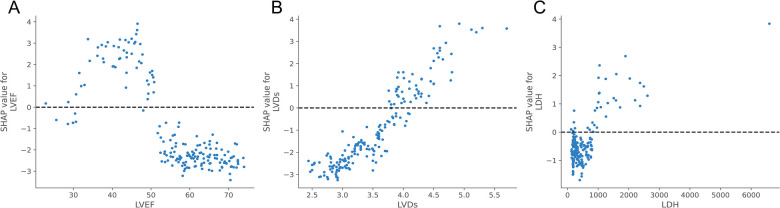
SHAP dependence plot of the XGBoost model. Each dependence plot shows how a single feature affects the output of the prediction model, and each dot represents a single patient.**(A)** LVEF, **(B)** LVDs, **(C)** LDH. The SHAP values for these features exceed zero, representing an increased risk of HF.

Meanwhile, to elucidate how each feature impacted the probability of HF in the ML model, we plotted SHAP dependency graphs for three key features: LVEF, LVDs, and LDH. As shown in Figures 6A–C, LVEF was below approximately 50%, LVDs were higher than about 4.0 cm, and LDH levels above nearly 650 u/L were associated with an increased risk of HF.

**Figure 7:**
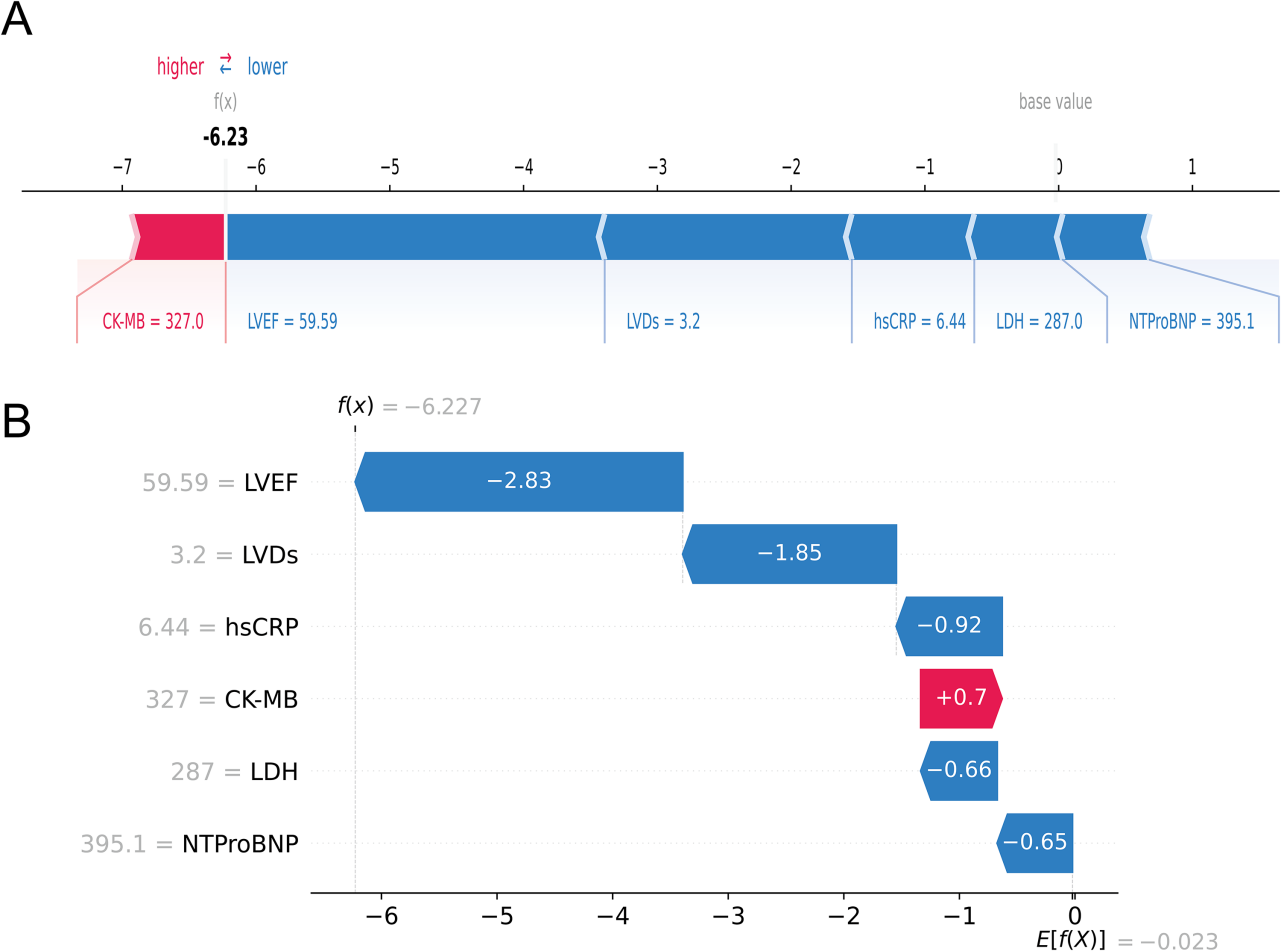
An AMI patient who did not experience HF during follow-up. The forceplot **(A)** and waterfall plot **(B)** are used to explain the contribution of features on a certain patient. wherein the red and blue bars signify risk factors and protective factors, respectively.

Next, at the individual level, we explained the personalized prediction results of two random samples through SHAP plots and waterfall plot analysis. The red and blue bars represented risk factors and protective factors, respectively. The length of each bar corresponded to its feature importance. In Figures 7A,B, we illustrated the case of an AMI patient who did not develop HF during the follow-up period. Notably, the presence of several protective factors, including normal LVEF (59.59%), LVDs (3.2 cm), hsCRP (6.44 mg/L), LDH (287 u/L), and NT-proBNP (395.1 pg/ml), led to the model predicting a relatively low risk (0.3%), consistent with the actual outcome (true negative), although CK-MB was relatively high (327 u/L).

**Figure 8:**
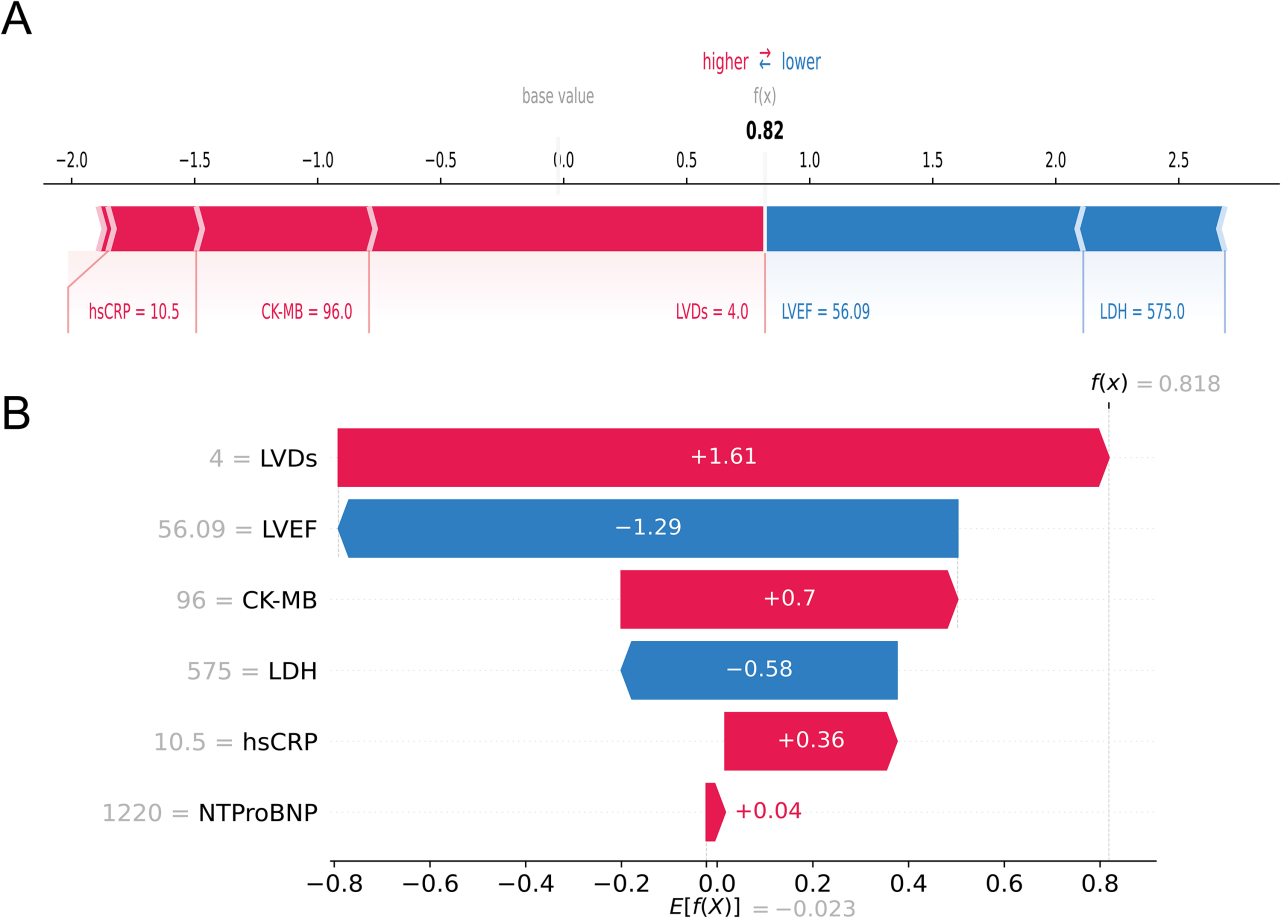
An AMI patient who developed HF during follow-up. The forceplot **(A)** and waterfall plot **(B)** are used to explain the contribution of features on a certain patient. wherein the red and blue bars signify risk factors and protective factors, respectively.

In contrast, Figures 8A,B depicted a case of an AMI patient who developed HF. Despite the patient's LVEF (56.09%) and LDH (575 u/L) being within normal ranges, the model forecasted a heightened probability of HF (69.3%) owing to the presence of multiple risk factors, notably larger LVDs (4.0 cm), elevated CK-MB (96 u/L), and heightened hsCRP (10.5 mg/L), which was consistent with the actual outcomes (true positive). Collectively, these individual-level elucidations aligned with the feature-level interpretations, offering a potential solution to the “black box” dilemma in medical AI applications.

## 4 Discussion

HF is a common and serious complication of myocardial infarction, which is closely related to high mortality and morbidity, regardless of the presence of coronary artery obstruction (23). Establishing a feasible prognosis prediction model was helpful for clinicians to distinguish high-risk patients and optimize the management of risk factors. To our knowledge, this study was the first to combine ML and SHAP methods to develop a HF risk prediction model for AMI patients. The main findings were as follows: (1) among the four algorithms tested, the XGBoost model performed optimal predictive power with the best discrimination and calibration; (2) the three most important variables associated with the incidence of HF after AMI included LVEF, LVDs, and LDH; and (3) SHAP method, revealed the roles of various variables in model interpretation and demonstrates the individual level prediction process. Moreover, the model utilized only a few easily accessible predictor variables, which enhances its utility in clinical applications.

ML was widely applied in clinical diagnosis and prognosis prediction (24–26). However, only a few studies used ML to predict HF after AMI. Li et al. employed LASSO, RF, and SVM-RFE algorithms to investigate genetic mechanisms of HF development after AMI, identifying IL1B, TIMP2, IFIT3, and P2RY2 as potential therapeutic targets (27). Additionally, Li et al. employed seven ML algorithms to predict the risk of HF after AMI based on clinical laboratory indicators, and found that XGBoost performed best with nine key indicators, including cTnI, TG, and URBC (14). However, these studies focused on limited factors and did not consider other potentially predictive indicators such as medication history, imaging, and coronary angiography. Therefore, more comprehensive exploration were needed to improve the prediction of HF after AMI.

Compared to previous studies (14, 27), our model encompassed a broader range of features. It used four common ML methods to predict the risk of HF in AMI patients within three years after PCI surgery, considering multiple factors such as demographics, clinical complications, laboratory tests, echocardiography parameters, and angiography results. The results showed that the XGBoost model had the highest discrimination ability, with an AUC of 0.922, an accuracy of 0.896, a sensitivity of 0.795, and a specificity of 0.939. XGBoost was an efficient ML method based on a scalable end-to-end tree boosting system, which processed large-scale data and high-dimensional feature (28, 29). It also used regularization techniques and pruning strategies to reduce the risk of overfitting (30). Additionally, the data source for this study comprised the initial test results of AMI patients upon admission, reflecting their initial health status. Therefore, the predictions had good foresight. This study also utilized hospital data, imposing no additional financial burden on patients, this underscored the potential of ML in clinical decisions.

Another advantage of our study was the introduction of the SHAP method for interpreting the XGBoost model. ML models were often referred to as black-box models because we cannot precisely understand the specific contribution of each feature to clinical decisions. Interpretability of a model can be defined as the extent to which a human can understand the cause of the ML model's prediction (31). The higher the interpretability of the model, the easier it will be for clinicians to understand the model's behavior and trust the model's conclusion, so as to make appropriate clinical decisions in the best interests of the patient (32). Therefore, we introduced the SHAP method to address the black-box problem. Based on game theory, SHAP clearly explained the complex relationships between features and prediction results, offering significant advantages in terms of interpretability and visualization. Additionally, we provided a ranking of characteristics for individual cases through SHAP analysis. Under the comprehensive influence of these variables, we can predict whether a person may suffer from “HF” or “Non-HF”. As shown in Figures 7, 8, with SHAP personalized analysis, the physician can intuitively understand how the ML model makes decisions, and therefore use the model for clinical decision-making.

Our research findings indicated that lower LVEF and larger LVDs predicted a higher risk of HF. LVEF and LVDs were indicators used to evaluate cardiac function and structure. In some patients, left ventricular remodeling caused by left ventricular myocardial repair and functional compensation 24–72 h after AMI aggravated the degree of myocardial injury, which could lead to the decrease of LVEF, left ventricular dilatation and malignant arrhythmia. As the disease progressed, it resulted in HF or even death (33). A multi-ethnic atherosclerosis study (MESA) found that during an average follow-up of 9.4 years, left ventricular remodeling was closely related to HF events. Compared with subjects with normal LV size and preserved LVEF, participants with left ventricular dilation and reduced LVEF had a worse prognosis (34). Furthermore, Michael et al. also demonstrated that subclinical left ventricular dilation and systolic dysfunction were independent predictors of HF (35).

Our research also indicated that elevated levels of LDH, NT-proBNP, and CK-MB are associated with an increased risk of HF in patients with AMI. As specific markers of myocardial injury, the levels of LDH, NT-proBNP, and CK-MB can reflect the severity of myocardial cell damage and deterioration of cardiac function. Numerous previous studies have confirmed that elevated levels of these markers are closely related to poor prognosis (36–40). Similarly, in our prediction model, hsCRP was considered one of the six key predictors for identifying HF risk in AMI patients. Higher levels of hsCRP in these patients may indicate a greater risk of HF, as it reflects immune system damage that can lead to severe complications. As a marker of inflammation severity, hsCRP is closely related to an increased risk of HF (41).

This study had some limitations. Firstly, this was a retrospective study, and there may be some causal inference and selection bias. Secondly, whilst our model was validated on an internal test dataset, external validation on another dataset would be ideal and necessary prior to consideration of widespread use, Future research will conduct large-scale multicenter clinical studies and develop an online prediction system to better support clinical application. Finally, our study mainly extracted the clinical data of AMI patients within 24 h after admission, which may ignore the dynamic changes of these characteristics with time.

## 5 Conclusion

This study successfully developed an interpretable machine learning model to predict the risk of HF in AMI patients. This model aids clinicians in tailoring individualized treatment strategies based on each patient's unique prognostic profile, thereby improving patient outcomes.

## Data Availability

The raw data supporting the conclusions of this article will be made available by the authors, without undue reservation.
